# Editorial: NeuroHaptics: From Human Touch to Neuroscience

**DOI:** 10.3389/fnins.2022.964014

**Published:** 2022-07-25

**Authors:** Wanjoo Park, Laehyun Kim, Tonio Ball, S. Farokh Atashzar

**Affiliations:** ^1^Engineering Division, New York University Abu Dhabi, Abu Dhabi, United Arab Emirates; ^2^Center for Bionics, Korea Institute of Science and Technology, Seoul, South Korea; ^3^Department of Neurosurgery, University Hospital Freiburg, Freiburg im Breisgau, Germany; ^4^Department of Electrical and Computer Engineering, New York University, New York, NY, United States

**Keywords:** haptics in AR/VR, tactile perception, tactile communication, brain-computer interface, motor rehabilitation

Haptics and Neuroscience have become integrated fields, allowing for an in-depth understanding of human sensorimotor capacity and functionality. There has been an accelerated surge in Neuroscience focusing on various modalities of haptics (including tactile and kinaesthetic) thanks to the advanced technologies applying various forms of haptic feedback, such as tactile internet and wearable haptics combined with virtual and augmented reality modules.

A challenge in conventional studies is the reliance on subjective self-report assessments, limiting the generalizability of the conclusions and increasing the uncertainty, variability, and susceptibility of the assessments to cognitive, memory, and communication barriers, and increasing the sensitivity to the timing of answer collection.

Therefore, taking advantage of the novel objective assessment of neural circuitry, more recent efforts are focused on processing central neural responses to shed light on the neurophysiology of haptics and to better understand the functionality of the human nervous system related to haptics. In this regard, the real-time measurement of electroencephalography and functional MRI has attracted a great deal of interest in recent studies to probe human neural functions during haptics exploration and experiments. This will also allow for a better understanding of haptics and subtle underlying mechanisms that could not be detected using subjective methods.

Our Research Topic consists of a total of seven papers divided into four categories. The first category is a challenge to the limitation of motor imagery-based brain-computer interface (MI-BCI), which is one of the most used methods in BCI, is difficult to detect in some people, and this leads to poor BCI performance. In a study to solve this problem, first, it was found that theta oscillation (4–8 Hz) in the eye-open resting state was correlated with MI-BCI performance (Kang et al.). It shows that the performance of MI-BCI can be improved through calibration using this. In a second study, a new method called somatosensory-MI was applied (Park et al.). It was possible to improve the performance of participants with low MI performance by more than 10% through training that combined motor execution and somatosensory sensation. It is expected that these studies will make a great contribution to solving the illiteracy or inefficient problem of MI-BCI and improving the performance of MI-BCI.

The second category is a study on the neuro-representation of tactile sensation. Traditionally, many studies on user response to tactile sensation have been conducted in the research area of haptics; however, there is not enough evidence for neuroscientific response to tactile sensation to explain the reason. Three papers present neuroscientific evidence through Electroencephalography (EEG) in perception studies on ridged texture (Tang et al.), laser-induced (Kim et al.), and different levels of intensities (Park et al.). In the first study, the perception of ridged surfaces was found in the early components of P100 and P200 and the late component of P300 through non-linear analysis methods. The second study shows that laser-evoked tactile sensation can be produced. Compared with sham stimulation, they reported that alpha and beta event-related desynchronization (ERD) are represented through the physical object and laser stimulation. The third study is on vibration, which is one of the most used haptic stimuli in the literature, while the corresponding standard is ambiguous about how much intensity vibration should be used for the stimuli. In addition, although the user's feeling varies according to the intensity of vibration, there was insufficient quantitative evidence on what kind of difference this makes in the brain. In this study, differences appeared in alpha and beta ERD and P200 and P300 event-related potential (ERP) components according to vibration intensity. In particular, the study shows that the intensity of vibration can be distinguished by how strong and how long the alpha ERD is. The aforementioned three studies aim to find the neuroscientific basis for various tactile sensations and to shed light on designing stimuli based on neuroscientific evidence in haptics.

In the third category, it shows that it is possible to classify the reaching-to-grasping tasks of the upper extremity surface electromyography signal through a conventional neural network (CNN) (Kim et al.). Reaching-to-grasping tasks are one of the most used functions in daily life and rehabilitation training. However, decoding this motion through bio-signals was a significant challenge. In this study, using principal component analysis-based CNN shows a significant improvement compared to the conventional methods. The fourth category is a study on how the use of head-mounted display (HMD) for virtual reality (VR) can affect the EEG signal (Weber et al.). Recently, VR environments have been used for conducting a wide range of neuroscientific studies. Therefore, it is necessary to consider the electromagnetic interference of digital devices such as HMDs on EEG signal. In this study, it was shown that two types of HMDs and 64-channel EEG were used without significant effect on EEG signals below 50 Hz. However, it shows that there is an effect in the high gamma band above 50 Hz.

This Research Topic aimed to collect the most recent novel efforts related to objective measurements of neural responses for haptics. The goal was to inform and further accelerate NeuroHaptics research in both the haptics and Neuroscience communities. In other words, this Research Topic is to promote activities in the haptics domain, focusing on an objective and in-depth neuroscientific understanding of various modalities of “touch”. We expect that this Research Topic is to promote activities in Neuroscience, focusing on the fusion of multisensory domains with a specific focus on haptics.

## Author contributions

WP drafted the manuscript. LK, TB, and SA critically revised the manuscript. All authors approved the submitted version.

## Funding

This work was supported in part by the Institute of Information & Communications Technology Planning & Evaluation (IITP), funded by the Korean government (MSIT) (Grant No. 2017- 000432), the NYUAD Center for Artificial Intelligence and Robotics (CAIR), funded by Tamkeen under the NYUAD Research Institute Award # CG010, US National Science Foundation, Award #: 2037878, and BrainLinks-BrainTools EXC1086.

## Conflict of interest

The authors declare that the research was conducted in the absence of any commercial or financial relationships that could be construed as a potential conflict of interest.

## Publisher's note

All claims expressed in this article are solely those of the authors and do not necessarily represent those of their affiliated organizations, or those of the publisher, the editors and the reviewers. Any product that may be evaluated in this article, or claim that may be made by its manufacturer, is not guaranteed or endorsed by the publisher.

